# (4-Benzyl-1-methyl-1,2,4-triazol-5-yl­idene)[(1,2,5,6-η)-cyclo­octa-1,5-diene](tri­phenyl­phosphane-κ*P*)iridium(I) tetra­fluorido­borate

**DOI:** 10.1107/S2414314621008361

**Published:** 2021-08-17

**Authors:** Elliott B. Newman, Andrei V. Astashkin, Daniel R. Albert, Edward Rajaseelan

**Affiliations:** a Department of Chemistry, Millersville University, Millersville PA, 17551, USA; b Department of Chemistry and Biochemistry, The University of Arizona, Tuscon, AZ, 85716, USA; Vienna University of Technology, Austria

**Keywords:** crystal structure, iridium, N-heterocyclic carbenes, cationic complexes

## Abstract

A new triazole-based N-heterocyclic carbene iridium(I) cationic complex with a tetra­fluorido­borate counter-anion, [Ir(C_10_H_11_N_3_)(C_8_H_12_)(C_18_H_15_P)]BF_4_, has been synthesized and structurally characterized. The cationic complex exhibits a distorted square-planar environment around the Ir^I^ ion. One significant non-standard hydrogen-bonding inter­action exists between a hydrogen atom on the N-heterocyclic carbene ligand and a fluorine atom from the counter-ion, BF_4_^−^. In the crystal, π–π stacking inter­actions are observed between one of the phenyl rings and the triazole ring. Both inter­molecular and intra­molecular C—H⋯π(ring) inter­actions are also observed.

## Abstract 2

The cationic complex in the title salt, [Ir(C_8_H_12_)(C_18_H_15_P)(C_10_H_11_N_3_)]BF_4_, exhibits a distorted square-planar geometry around the Ir^I^ ion.

## Graphical abstract

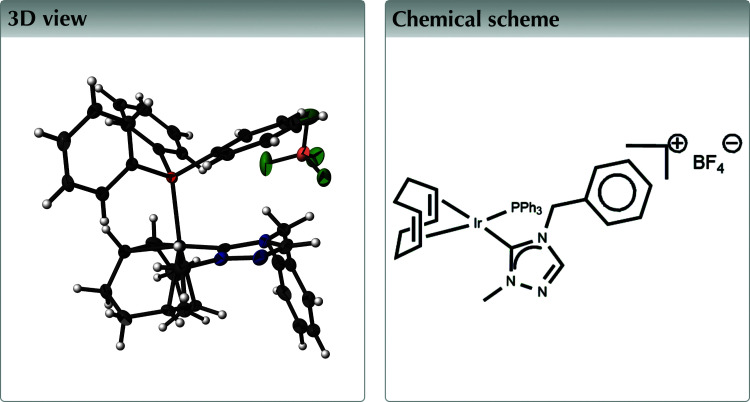

## Structure description

Transition-metal complexes containing N-heterocyclic carbene (NHC) ligands are of inter­est for their many useful applications in synthesis and catalysis (Díez-González *et al.*, 2009 ▸; Herrmann, 2002 ▸; Ruff *et al.*, 2016 ▸; Zuo *et al.*, 2014 ▸; Albrecht *et al.*, 2002 ▸; Gnanamgari *et al.*, 2007 ▸). The NHC ligands can be tuned sterically and electronically by having different substituents on the nitro­gen atoms (Gusev, 2009 ▸). Many imidazole- and triazole-based NHC rhodium and iridium complexes have been synthesized and structurally characterized (Herrmann *et al.*, 2006 ▸; Wang & Lin, 1998 ▸; Chianese *et al.*, 2004 ▸; Nichol *et al.*, 2009 ▸, 2010 ▸, 2011 ▸, 2012 ▸; Idrees *et al.*, 2017*a* ▸,*b* ▸; Rood *et al.*, 2021 ▸).

**Figure 1:**
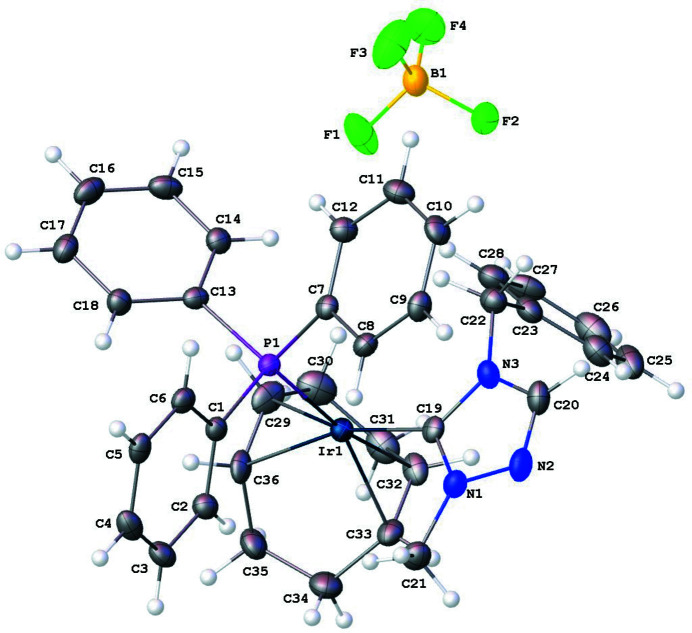
The mol­ecular entities in the crystal structure of the title compound (**4**). Displacement ellipsoids are drawn at the 50% probability level.

The mol­ecular structure of the title salt, [Ir(C_10_H_11_N_3_)(C_8_H_12_)(C_18_H_15_P)]BF_4_ (**4**), comprises an Ir^I^ cation complex and a tetra­fluorido­borate counter-anion, illustrated in Fig. 1 ▸. The coordination environment around the Ir^I^ ion, formed by the bidentate cyclo­octa-1,5-diene (COD), NHC, and tri­phenyl­phosphane ligands, results in a distorted square-planar environment. The Ir—C19(NHC) bond length is 2.039 (3) Å. The carbene(C19)—Ir—P bond angle is 89.52 (9)°. The carbene atom, C19, deviates from the expected bond angle of an *sp* ^2^ hybridized atom with an N1—C19—N3 angle of 102.6 (3)°.

**Figure 2:**
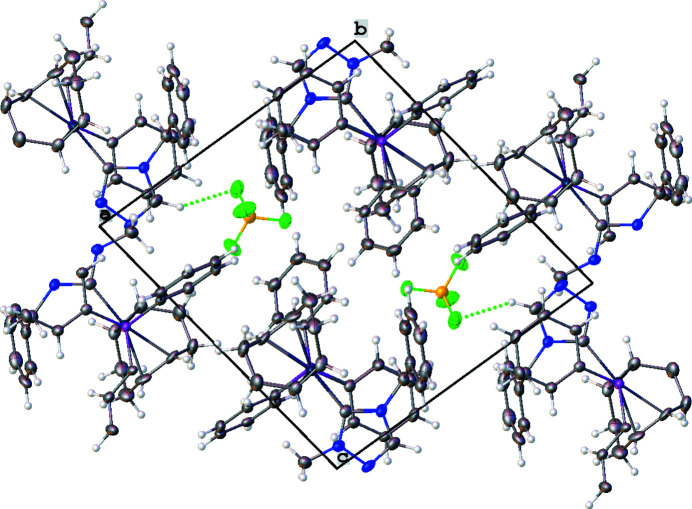
Crystal packing unit-cell diagram of the title compound (**4**). Non-covalent inter­actions are shown as dotted green lines.

**Figure 3:**
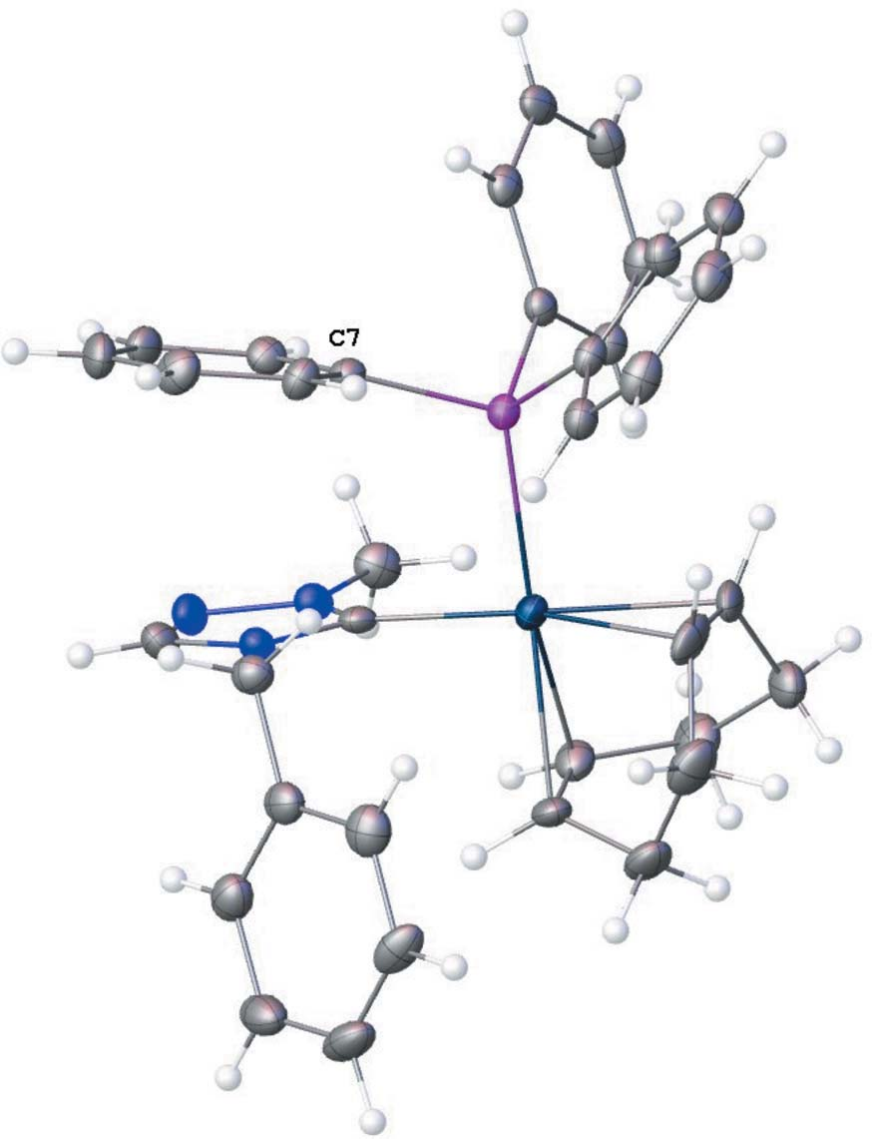
View of the title compound (**4**) showing a distorted inter­molecular parallel inter­action between a phenyl ring (C7) of the tri­phenyl­phosphane ligand and the NHC ring.

**Figure 4:**
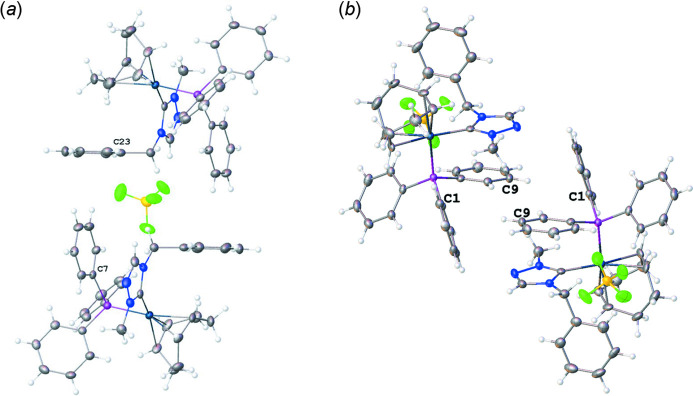
Views of inter­molecular inter­actions of the title compound (**4**) showing T-shaped, distorted perpendicular inter­actions. (*a*) View of the near perpendicular orientation of a phenyl ring (C7) of the tri­phenyl­phosphane ligand on one moiety and the phenyl ring (C23) attached to the NHC ligand; (*b*) view of distorted perpendicular arranged phenyl rings that are influenced by the C9—H9⋯π[phenyl ring(C1)] inter­molecular inter­actions.

Fig. 2 ▸ shows the crystal packing of the complex. There is one non-covalent F⋯H inter­action between F2 of the tetra­fluorido­borate anion and H20, which is connected to C20(NHC), that is significantly shorter than the sum of the van der Waals radii (Fig. 2 ▸, Table 1 ▸). An intra­molecular distorted parallel π–π stacking inter­action is observed between the triazole ring and one of the phenyl rings (C7–C12) at the phosphane (Fig. 3 ▸) with an inter­centroid distance of 3.682 (2) Å and a slippage of 1.584 Å. The dihedral angle between the triazole and the phenyl phosphane ring planes is 13.0 (2)°. Both intra­molecular and inter­molecular C—H⋯π(ring) inter­actions impact the orientations of phenyl rings. The COD ligand and the phenyl wingtip of the triazole are oriented *via* an intra­molecular C32—H32⋯π [phenyl wingtip of triazole; (C23–C28)] inter­action that has an H⋯centroid distance of 2.88 Å and a C—H⋯centroid angle of 133°. Inter­molecular, distorted perpendicular T-shaped orientations are observed between phenyl rings (Fig. 4 ▸). The (C7–C12) ring at the phosphane and the wingtip (C23–C28) phenyl ring show a nearly perpendicular orientation (Fig. 4 ▸ *a*) with a dihedral angle between the two ring planes of 85.98 (18)°; however, this orientation is not directly associated with C—H⋯π inter­actions. An inter­molecular C9—H9⋯π[phenyl C1(phosphane)] inter­action has an H⋯centroid distance of 2.77 Å and a C—H⋯centroid angle of 153° (Fig. 4 ▸ *b*).

## Synthesis and crystallization

**Figure 5:**
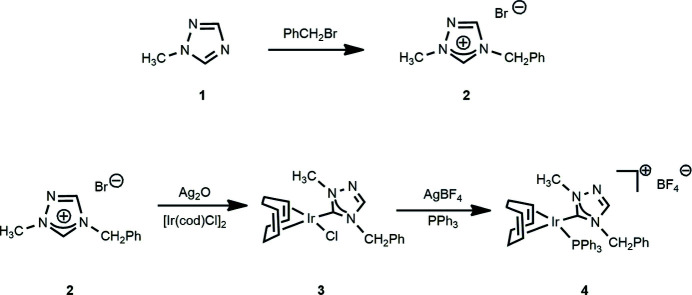
Reaction scheme showing the synthesis of the N-heterocyclic carbene (**2**) and the subsequent formation of the title ionic compound (**4**).

1-Methyl triazole (**1**) was purchased from Matrix Scientific. All other compounds used in the syntheses as shown in Fig. 5 ▸ were obtained from Sigma–Aldrich and Strem and used as received; all syntheses were performed under a nitro­gen atmosphere. NMR spectra were recorded at room temperature in CDCl_3_ on a 400 MHz (operating at 162 MHz for ^31^P) Varian spectrometer and referenced to the residual solvent peak (δ in ppm).

**1-Methyl-4-benzyl-1,2,4-triazolium bromide (2)**: 1-Methyl-1,2,4-triazole (**1**) (1.230 g, 14.80 mmol) and benzyl bromide (5.010 g, 29.29 mmol) were added to toluene (10 ml) and the mixture was refluxed for 48 h. Once cooled, ether was added and the product was filtered off as a white powder, yield: 2.78 g (57%). ^1^H NMR: δ 11.62 (*s*, 1 H, N—C_5_H—N), 8.71 (*s*, 1 H, N—C_3_—N), 7.62–7.60 (*m*, 2 H, H_arom_), 7.15–7.26 (*m*, 3 H, H_arom_), 5.83 (*s*, 2 H, CH_2_Ph), 4.22 (*s*, 3 H, CH_3_) ^13^C NMR: δ 143.52 (N—CH—N), 142.65 (N—CH—N), 131.56, 130.09, 129.79, 129.40 (C_arom_), 52.32 (CH_2_Ph), 39.62 (CH_3_).

**[(1,2,5,6-η)-Cyclo­octa-1,5-diene](1-methyl-4-benzyl-1,2,4-triazol-5-yl­idene)chloro­iridium (3)**: Triazolium bromide (**2**) (51.92 mg, 0.298 mmol) and Ag_2_O (34.53 mg, 0.149 mmol) were stirred under dark conditions for 1.5 h in CH_2_Cl_2_ (10 ml). The mixture was then filtered through Celite into [Ir(COD)Cl]_2_ (100 mg, 0.149 mmol) and stirred in the dark for 1.5 h. The resulting solution was filtered through Celite and the solvent was removed under reduced pressure. The orange solid product (**3**) was placed under vacuum to dry, yield: 132 mg (100%). ^1^H NMR: δ 7.71 (*s*, 1 H, N–C_3_H–N), 7.38–7.31 (*m*, 5 H, H_arom_), 5.68 (*m*, 2 H, CH_COD_), 5.29 (*s*, 2 H, CH_2_Ph), 4.73 (*m*, 2 H, CH_COD_), 4.14 (*s*, 3 H, CH_3_), 3.03–2.75 [*m*, 2 H, (CH_2_)_COD_], 2.25 [*m*, 2 H, (CH_2_)_COD_], 2.10 [*m*, 2 H, (CH_2_)_COD_], 1.98–1.85 [*m*, 2 H, (CH_2_)_COD_]. ^13^C NMR: δ 183.13 (Ir—C), 141.63 (N—C_3_H—N), 129.20, 128.99, 128.79, 128.45 (C_arom_), 87.05, 86.71, 52.10, 52.05 (CH_COD_), 52.69 (CH_2_Ph), 39.60 (CH_3_), 33.80, 33.13, 29.68, 29.15 (CH_2_)_COD_.

**(4-Benzyl-1-methyl-1,2,4-triazol-5-yl­idene)[(1,2,5,6-η)-cyclo­octa-1,5-diene](tri­phenyl­phosphane-κ*P*)iridium(I) tetra­fluorido­borate (4)**: Tri­phenyl­phosphane (80.8 mg, 0.308 mmol) and AgBF_4_ (59.95 mg, 0.308 mmol) were added directly to (**3**) (132 mg, 0.308 mmol) in CH_2_Cl_2_ (10 ml). The solution was stirred under dark conditions for 1.5 h. The mixture was filtered through Celite, and the solvent was removed under reduced pressure. The bright-orange solid product (**4**) was dried under vacuum, yield: 210 mg (91.7%). ^1^H NMR: δ 7.89 (*s*, 1 H, N—C_3_H—N) 7.53–7.26 (*m*, 20 H, H_arom_), 5.29 (*s*, 2 H, CH_2_Ph), 5.38, 5.34, 4.85, 4.82 (*m*, 4 H, CH_COD_), 3.69 (*s*, 3 H, CH_3_), 2.33 [*m*, 5 H, (CH_2_)_COD_], 2.08 [*m*, 3 H, (CH_2_)_COD_]. ^13^C NMR: δ 179.25 (Ir—C), 143.65 (N—C_3_H—N), 134.23–133.71 (C_arom_), 88.28, 88.17, 86.24, 86.12 (CH_COD_), 52.02 (CH_2_Ph), 39.76 (CH_3_), 31.97, 31.44, 30.24, 29.82 (CH_2_)_COD_. ^31^P NMR: δ 17.08.

The title compound (**4**) was crystallized by slow diffusion of pentane into a CH_2_Cl_2_ solution.

## Refinement

Crystal data, data collection and structure refinement details are summarized in Table 2 ▸.

## Supplementary Material

Crystal structure: contains datablock(s) I. DOI: 10.1107/S2414314621008361/wm4152sup1.cif

Structure factors: contains datablock(s) I. DOI: 10.1107/S2414314621008361/wm4152Isup2.hkl

CCDC reference: 2102850

Additional supporting information:  crystallographic information; 3D view; checkCIF report

## Figures and Tables

**Table 1:** Hydrogen-bond geometry (Å, °)

*D*—H⋯*A*	*D*—H	H⋯*A*	*D*⋯*A*	*D*—H⋯*A*
C20—H20⋯F2^i^	0.95	2.29	3.131 (4)	147

Symmetry code: (i) 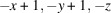 .

**Table 2:** Experimental details

Crystal data	
Chemical formula	[Ir(C_10_H_11_N_3_)(C_8_H_12_)(C_18_H_15_P)]BF_4_
*M*_r_	822.67
Crystal system, space group	Triclinic, *P* 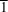
Temperature (K)	100
*a*, *b*, *c* (Å)	10.7158 (17), 13.075 (2), 13.2554 (19)
α, β, γ (°)	77.680 (5), 78.110 (5), 67.114 (6)
*V* (Å^3^)	1655.9 (4)
*Z*	2
Radiation type	Mo *K*α
μ (mm^−1^)	4.13
Crystal size (mm)	0.20 × 0.09 × 0.04

Data collection	
Diffractometer	Bruker APEXII CCD
Absorption correction	Multi-scan (*SADABS*; Krause *et al.*, 2015 ▸)
*T*_min_, *T*_max_	0.596, 0.745
No. of measured, independent and observed [*I* > 2σ(*I*)] reflections	67109, 6342, 5672
*R*_int_	0.059
(sin θ/λ)_max_ (Å^−1^)	0.612

Refinement	
*R*[*F* ^2^ > 2σ(*F* ^2^)], *wR*(*F* ^2^), *S*	0.023, 0.052, 1.05
No. of reflections	6342
No. of parameters	416
H-atom treatment	H-atom parameters constrained
Δρ_max_, Δρ_min_ (e Å^−3^)	1.26, −0.46

Computer programs: *SAINT* (Bruker, 2013 ▸), *APEX2* (Bruker, 2013 ▸), *SHELXT* (Sheldrick, 2015*a* ▸), *SHELXL* (Sheldrick, 2015*b* ▸), and *OLEX2* (Dolomanov *et al.*, 2009 ▸).

## full crystallographic data

### Crystal data

**Table:**

[Ir(C_10_H_11_N_3_)(C_8_H_12_)(C_18_H_15_P)]BF_4_	*Z* = 2
*M**_r_* = 822.67	*F*(000) = 816
Triclinic, *P*1	*D*_x_ = 1.650 Mg m^−^^3^
*a* = 10.7158 (17) Å	Mo *K*α radiation, λ = 0.71073 Å
*b* = 13.075 (2) Å	Cell parameters from 9445 reflections
*c* = 13.2554 (19) Å	θ = 2.4–25.7°
α = 77.680 (5)°	µ = 4.13 mm^−^^1^
β = 78.110 (5)°	*T* = 100 K
γ = 67.114 (6)°	Plate, clear light red
*V* = 1655.9 (4) Å^3^	0.20 × 0.09 × 0.04 mm

### Data collection

**Table:**

Bruker APEXII CCD diffractometer	5672 reflections with *I* > 2σ(*I*)
φ and ω scans	*R*_int_ = 0.059
Absorption correction: multi-scan (*SADABS*; Krause *et al.*, 2015)	θ_max_ = 25.8°, θ_min_ = 1.6°
*T*_min_ = 0.596, *T*_max_ = 0.745	*h* = −13→13
67109 measured reflections	*k* = −15→15
6342 independent reflections	*l* = −16→16

### Refinement

**Table:**

Refinement on *F*^2^	0 restraints
Least-squares matrix: full	Hydrogen site location: inferred from neighbouring sites
*R*[*F*^2^ > 2σ(*F*^2^)] = 0.023	H-atom parameters constrained
*wR*(*F*^2^) = 0.052	*w* = 1/[σ^2^(*F*_o_^2^) + (0.0247*P*)^2^ + 1.1818*P*] where *P* = (*F*_o_^2^ + 2*F*_c_^2^)/3
*S* = 1.05	(Δ/σ)_max_ = 0.003
6342 reflections	Δρ_max_ = 1.26 e Å^−^^3^
416 parameters	Δρ_min_ = −0.46 e Å^−^^3^

### Special details

**Table:**

Geometry. All esds (except the esd in the dihedral angle between two l.s. planes) are estimated using the full covariance matrix. The cell esds are taken into account individually in the estimation of esds in distances, angles and torsion angles; correlations between esds in cell parameters are only used when they are defined by crystal symmetry. An approximate (isotropic) treatment of cell esds is used for estimating esds involving l.s. planes.

### Fractional atomic coordinates and isotropic or equivalent isotropic displacement parameters (Å^2^)

**Table:**

	*x*	*y*	*z*	*U*_iso_*/*U*_eq_	
Ir1	0.34407 (2)	0.83541 (2)	0.28200 (2)	0.01759 (5)	
P1	0.12536 (8)	0.83532 (7)	0.28916 (6)	0.01633 (17)	
F2	0.4434 (2)	0.39812 (19)	0.15141 (17)	0.0457 (6)	
F1	0.3358 (3)	0.43725 (18)	0.31219 (16)	0.0488 (6)	
F4	0.4370 (2)	0.25669 (18)	0.2837 (2)	0.0513 (6)	
F3	0.2451 (2)	0.3691 (2)	0.21395 (18)	0.0550 (7)	
N1	0.3451 (3)	0.9438 (2)	0.0525 (2)	0.0207 (6)	
N3	0.4149 (3)	0.7670 (2)	0.0628 (2)	0.0203 (6)	
N2	0.3729 (3)	0.9230 (2)	−0.0497 (2)	0.0254 (6)	
C19	0.3684 (3)	0.8500 (3)	0.1236 (2)	0.0183 (7)	
C7	0.1030 (3)	0.7845 (3)	0.1776 (2)	0.0179 (7)	
C32	0.5665 (3)	0.7983 (3)	0.2821 (3)	0.0253 (8)	
H32	0.626600	0.768329	0.218537	0.030*	
C12	0.1238 (3)	0.6723 (3)	0.1783 (2)	0.0216 (7)	
H12	0.132883	0.622109	0.242116	0.026*	
C1	−0.0069 (3)	0.9751 (3)	0.2855 (2)	0.0178 (7)	
C14	0.1431 (3)	0.6395 (3)	0.4289 (2)	0.0214 (7)	
H14	0.228932	0.607976	0.388206	0.026*	
C13	0.0628 (3)	0.7527 (3)	0.4041 (2)	0.0185 (7)	
C17	−0.1060 (4)	0.7313 (3)	0.5514 (3)	0.0259 (8)	
H17	−0.190698	0.762792	0.593455	0.031*	
C8	0.0849 (3)	0.8575 (3)	0.0824 (2)	0.0203 (7)	
H8	0.068820	0.934669	0.080199	0.024*	
C3	−0.0835 (4)	1.1709 (3)	0.3063 (2)	0.0245 (8)	
H3	−0.064632	1.231798	0.319269	0.029*	
C9	0.0902 (3)	0.8179 (3)	−0.0080 (2)	0.0227 (7)	
H9	0.076555	0.868303	−0.071510	0.027*	
C2	0.0192 (3)	1.0652 (3)	0.3053 (2)	0.0218 (7)	
H2	0.107875	1.054315	0.318214	0.026*	
C36	0.3090 (4)	0.8736 (3)	0.4425 (2)	0.0252 (8)	
H36	0.210418	0.902550	0.472981	0.030*	
C24	0.7091 (4)	0.6176 (3)	0.0575 (3)	0.0295 (8)	
H24	0.691006	0.663040	−0.007934	0.035*	
C15	0.0973 (4)	0.5734 (3)	0.5127 (3)	0.0274 (8)	
H15	0.151318	0.496286	0.528467	0.033*	
C16	−0.0268 (4)	0.6188 (3)	0.5739 (3)	0.0290 (8)	
H16	−0.057365	0.572776	0.631137	0.035*	
C18	−0.0605 (3)	0.7981 (3)	0.4664 (2)	0.0217 (7)	
H18	−0.114496	0.875358	0.451064	0.026*	
C27	0.7615 (4)	0.4819 (3)	0.2459 (3)	0.0293 (8)	
H27	0.780485	0.433562	0.309845	0.035*	
C33	0.4969 (3)	0.9138 (3)	0.2672 (3)	0.0249 (8)	
H33	0.517503	0.951417	0.195153	0.030*	
C4	−0.2130 (4)	1.1872 (3)	0.2885 (2)	0.0256 (8)	
H4	−0.283208	1.259121	0.289832	0.031*	
C5	−0.2409 (3)	1.0984 (3)	0.2686 (2)	0.0219 (7)	
H5	−0.330069	1.109959	0.256515	0.026*	
C6	−0.1389 (3)	0.9931 (3)	0.2665 (2)	0.0210 (7)	
H6	−0.158122	0.933056	0.252059	0.025*	
C10	0.1153 (3)	0.7053 (3)	−0.0066 (3)	0.0264 (8)	
H10	0.121440	0.678008	−0.069057	0.032*	
C20	0.4150 (3)	0.8144 (3)	−0.0389 (3)	0.0256 (8)	
H20	0.442805	0.772812	−0.095430	0.031*	
C23	0.6015 (3)	0.5949 (3)	0.1266 (3)	0.0253 (8)	
C22	0.4580 (3)	0.6446 (3)	0.1003 (3)	0.0235 (7)	
H22A	0.451509	0.606348	0.045687	0.028*	
H22B	0.394730	0.630962	0.162938	0.028*	
C21	0.2877 (4)	1.0604 (3)	0.0705 (3)	0.0276 (8)	
H21A	0.352350	1.097757	0.036739	0.041*	
H21B	0.270688	1.063087	0.145552	0.041*	
H21C	0.201476	1.098840	0.041147	0.041*	
C35	0.3892 (4)	0.9431 (3)	0.4535 (3)	0.0304 (8)	
H35A	0.461895	0.896298	0.497187	0.036*	
H35B	0.327228	1.006922	0.490035	0.036*	
C11	0.1313 (4)	0.6327 (3)	0.0871 (3)	0.0269 (8)	
H11	0.147433	0.555611	0.088851	0.032*	
C34	0.4544 (4)	0.9885 (3)	0.3492 (3)	0.0331 (9)	
H34A	0.388607	1.062463	0.322974	0.040*	
H34B	0.535827	1.000569	0.360682	0.040*	
C31	0.6165 (4)	0.7308 (3)	0.3840 (3)	0.0317 (9)	
H31A	0.636207	0.779355	0.421715	0.038*	
H31B	0.702975	0.667823	0.368360	0.038*	
C29	0.3652 (4)	0.7579 (3)	0.4454 (3)	0.0305 (8)	
H29	0.298959	0.719362	0.477433	0.037*	
C28	0.6297 (4)	0.5259 (3)	0.2215 (3)	0.0305 (8)	
H28	0.558353	0.509268	0.269137	0.037*	
C25	0.8396 (4)	0.5758 (3)	0.0823 (3)	0.0356 (9)	
H25	0.910996	0.592979	0.035168	0.043*	
C30	0.5129 (4)	0.6837 (3)	0.4547 (3)	0.0379 (10)	
H30A	0.529545	0.609086	0.437450	0.045*	
H30B	0.528250	0.673106	0.528022	0.045*	
C26	0.8661 (4)	0.5072 (3)	0.1787 (3)	0.0360 (9)	
H26	0.955689	0.478381	0.197524	0.043*	
B1	0.3653 (4)	0.3650 (3)	0.2407 (3)	0.0281 (9)	

### Atomic displacement parameters (Å^2^)

**Table:**

	*U*^11^	*U*^22^	*U*^33^	*U*^12^	*U*^13^	*U*^23^
Ir1	0.01833 (7)	0.02064 (7)	0.01524 (7)	−0.00768 (5)	−0.00406 (5)	−0.00269 (5)
P1	0.0179 (4)	0.0173 (4)	0.0148 (4)	−0.0073 (3)	−0.0025 (3)	−0.0024 (3)
F2	0.0573 (15)	0.0482 (14)	0.0381 (13)	−0.0324 (12)	0.0197 (11)	−0.0193 (11)
F1	0.0662 (17)	0.0370 (13)	0.0301 (12)	−0.0019 (12)	−0.0027 (11)	−0.0138 (10)
F4	0.0429 (14)	0.0252 (12)	0.0688 (17)	0.0010 (11)	−0.0017 (12)	−0.0032 (11)
F3	0.0442 (15)	0.088 (2)	0.0366 (13)	−0.0345 (14)	−0.0070 (11)	0.0061 (13)
N1	0.0186 (14)	0.0229 (15)	0.0180 (14)	−0.0061 (12)	−0.0021 (11)	−0.0005 (11)
N3	0.0175 (14)	0.0228 (15)	0.0179 (14)	−0.0035 (12)	−0.0021 (11)	−0.0049 (11)
N2	0.0236 (15)	0.0374 (18)	0.0144 (14)	−0.0113 (14)	−0.0005 (11)	−0.0034 (12)
C19	0.0106 (15)	0.0245 (18)	0.0196 (16)	−0.0064 (13)	−0.0019 (13)	−0.0032 (14)
C7	0.0135 (15)	0.0224 (17)	0.0185 (16)	−0.0072 (13)	−0.0026 (12)	−0.0031 (13)
C32	0.0130 (16)	0.037 (2)	0.0281 (18)	−0.0064 (15)	−0.0045 (14)	−0.0129 (15)
C12	0.0253 (18)	0.0220 (17)	0.0194 (16)	−0.0091 (14)	−0.0073 (14)	−0.0018 (13)
C1	0.0199 (17)	0.0190 (17)	0.0136 (15)	−0.0070 (13)	−0.0014 (13)	−0.0016 (12)
C14	0.0248 (18)	0.0242 (18)	0.0168 (16)	−0.0095 (15)	−0.0038 (13)	−0.0041 (13)
C13	0.0249 (18)	0.0190 (17)	0.0148 (15)	−0.0107 (14)	−0.0065 (13)	−0.0002 (13)
C17	0.0262 (19)	0.034 (2)	0.0208 (17)	−0.0163 (16)	0.0002 (14)	−0.0044 (15)
C8	0.0205 (17)	0.0216 (17)	0.0197 (16)	−0.0101 (14)	−0.0016 (13)	−0.0010 (13)
C3	0.032 (2)	0.0219 (18)	0.0202 (17)	−0.0085 (15)	−0.0039 (15)	−0.0074 (14)
C9	0.0218 (18)	0.0299 (19)	0.0150 (16)	−0.0087 (15)	−0.0036 (13)	−0.0006 (14)
C2	0.0247 (18)	0.0228 (18)	0.0190 (16)	−0.0090 (14)	−0.0037 (14)	−0.0036 (13)
C36	0.0250 (18)	0.043 (2)	0.0109 (15)	−0.0148 (16)	0.0001 (13)	−0.0082 (14)
C24	0.030 (2)	0.028 (2)	0.0290 (19)	−0.0052 (16)	−0.0065 (16)	−0.0095 (15)
C15	0.041 (2)	0.0196 (18)	0.0247 (18)	−0.0127 (16)	−0.0126 (16)	0.0010 (14)
C16	0.040 (2)	0.036 (2)	0.0178 (17)	−0.0239 (18)	−0.0052 (16)	0.0031 (15)
C18	0.0247 (18)	0.0231 (18)	0.0166 (16)	−0.0084 (14)	−0.0027 (14)	−0.0023 (13)
C27	0.034 (2)	0.0157 (17)	0.038 (2)	−0.0018 (15)	−0.0210 (17)	−0.0021 (15)
C33	0.0254 (19)	0.034 (2)	0.0228 (17)	−0.0188 (16)	−0.0012 (14)	−0.0067 (15)
C4	0.032 (2)	0.0196 (18)	0.0181 (17)	−0.0015 (15)	−0.0040 (14)	−0.0017 (13)
C5	0.0211 (17)	0.0265 (18)	0.0130 (15)	−0.0064 (15)	−0.0013 (13)	0.0021 (13)
C6	0.0258 (18)	0.0242 (18)	0.0140 (16)	−0.0119 (15)	−0.0016 (13)	−0.0001 (13)
C10	0.0295 (19)	0.033 (2)	0.0207 (17)	−0.0122 (16)	−0.0027 (15)	−0.0118 (15)
C20	0.0189 (17)	0.035 (2)	0.0193 (17)	−0.0065 (15)	−0.0002 (14)	−0.0062 (15)
C23	0.0242 (18)	0.0250 (19)	0.0290 (19)	−0.0075 (15)	−0.0024 (15)	−0.0126 (15)
C22	0.0213 (18)	0.0225 (18)	0.0265 (18)	−0.0067 (14)	−0.0035 (14)	−0.0056 (14)
C21	0.0285 (19)	0.0248 (19)	0.0276 (19)	−0.0092 (16)	−0.0041 (15)	−0.0003 (15)
C35	0.031 (2)	0.038 (2)	0.0280 (19)	−0.0140 (17)	0.0000 (16)	−0.0173 (16)
C11	0.030 (2)	0.0226 (18)	0.0320 (19)	−0.0102 (15)	−0.0076 (16)	−0.0074 (15)
C34	0.038 (2)	0.034 (2)	0.037 (2)	−0.0190 (18)	−0.0045 (17)	−0.0126 (17)
C31	0.0222 (19)	0.034 (2)	0.036 (2)	−0.0003 (16)	−0.0154 (16)	−0.0083 (17)
C29	0.039 (2)	0.044 (2)	0.0149 (17)	−0.0227 (18)	−0.0137 (15)	0.0059 (15)
C28	0.037 (2)	0.0237 (19)	0.032 (2)	−0.0105 (16)	−0.0053 (16)	−0.0071 (15)
C25	0.024 (2)	0.048 (2)	0.043 (2)	−0.0145 (18)	0.0019 (17)	−0.0251 (19)
C30	0.044 (2)	0.034 (2)	0.035 (2)	−0.0083 (19)	−0.0227 (19)	0.0001 (17)
C26	0.023 (2)	0.036 (2)	0.050 (2)	0.0000 (17)	−0.0159 (18)	−0.0173 (19)
B1	0.028 (2)	0.027 (2)	0.024 (2)	−0.0063 (18)	0.0014 (17)	−0.0040 (17)

### Geometric parameters (Å, º)

**Table:**

Ir1—P1	2.3264 (9)	C36—C29	1.389 (5)
Ir1—C19	2.039 (3)	C24—H24	0.9500
Ir1—C32	2.241 (3)	C24—C23	1.403 (5)
Ir1—C36	2.212 (3)	C24—C25	1.372 (5)
Ir1—C33	2.204 (3)	C15—H15	0.9500
Ir1—C29	2.204 (3)	C15—C16	1.387 (5)
P1—C7	1.837 (3)	C16—H16	0.9500
P1—C1	1.823 (3)	C18—H18	0.9500
P1—C13	1.838 (3)	C27—H27	0.9500
F2—B1	1.394 (5)	C27—C28	1.381 (5)
F1—B1	1.385 (5)	C27—C26	1.383 (5)
F4—B1	1.382 (5)	C33—H33	1.0000
F3—B1	1.383 (5)	C33—C34	1.499 (5)
N1—N2	1.387 (4)	C4—H4	0.9500
N1—C19	1.349 (4)	C4—C5	1.392 (5)
N1—C21	1.458 (4)	C5—H5	0.9500
N3—C19	1.364 (4)	C5—C6	1.387 (4)
N3—C20	1.358 (4)	C6—H6	0.9500
N3—C22	1.485 (4)	C10—H10	0.9500
N2—C20	1.297 (4)	C10—C11	1.390 (5)
C7—C12	1.394 (4)	C20—H20	0.9500
C7—C8	1.408 (4)	C23—C22	1.502 (5)
C32—H32	1.0000	C23—C28	1.395 (5)
C32—C33	1.390 (5)	C22—H22A	0.9900
C32—C31	1.520 (5)	C22—H22B	0.9900
C12—H12	0.9500	C21—H21A	0.9800
C12—C11	1.389 (5)	C21—H21B	0.9800
C1—C2	1.399 (4)	C21—H21C	0.9800
C1—C6	1.408 (4)	C35—H35A	0.9900
C14—H14	0.9500	C35—H35B	0.9900
C14—C13	1.399 (4)	C35—C34	1.526 (5)
C14—C15	1.384 (5)	C11—H11	0.9500
C13—C18	1.389 (4)	C34—H34A	0.9900
C17—H17	0.9500	C34—H34B	0.9900
C17—C16	1.385 (5)	C31—H31A	0.9900
C17—C18	1.399 (4)	C31—H31B	0.9900
C8—H8	0.9500	C31—C30	1.532 (5)
C8—C9	1.387 (4)	C29—H29	1.0000
C3—H3	0.9500	C29—C30	1.515 (5)
C3—C2	1.393 (5)	C28—H28	0.9500
C3—C4	1.382 (5)	C25—H25	0.9500
C9—H9	0.9500	C25—C26	1.407 (5)
C9—C10	1.387 (5)	C30—H30A	0.9900
C2—H2	0.9500	C30—H30B	0.9900
C36—H36	1.0000	C26—H26	0.9500
C36—C35	1.518 (5)		
			
C19—Ir1—P1	89.52 (9)	C28—C27—C26	121.0 (3)
C19—Ir1—C32	92.03 (12)	C26—C27—H27	119.5
C19—Ir1—C36	162.76 (13)	Ir1—C33—H33	113.9
C19—Ir1—C33	89.07 (12)	C32—C33—Ir1	73.19 (19)
C19—Ir1—C29	159.50 (13)	C32—C33—H33	113.9
C32—Ir1—P1	168.59 (9)	C32—C33—C34	125.5 (3)
C36—Ir1—P1	94.42 (9)	C34—C33—Ir1	108.8 (2)
C36—Ir1—C32	87.42 (12)	C34—C33—H33	113.9
C33—Ir1—P1	154.95 (9)	C3—C4—H4	119.9
C33—Ir1—C32	36.44 (13)	C3—C4—C5	120.2 (3)
C33—Ir1—C36	80.27 (12)	C5—C4—H4	119.9
C33—Ir1—C29	94.84 (13)	C4—C5—H5	119.9
C29—Ir1—P1	95.18 (10)	C6—C5—C4	120.2 (3)
C29—Ir1—C32	79.59 (13)	C6—C5—H5	119.9
C29—Ir1—C36	36.67 (13)	C1—C6—H6	119.9
C7—P1—Ir1	113.78 (10)	C5—C6—C1	120.2 (3)
C7—P1—C13	104.54 (14)	C5—C6—H6	119.9
C1—P1—Ir1	113.37 (11)	C9—C10—H10	120.3
C1—P1—C7	102.82 (14)	C9—C10—C11	119.4 (3)
C1—P1—C13	104.00 (14)	C11—C10—H10	120.3
C13—P1—Ir1	116.84 (10)	N3—C20—H20	124.0
N2—N1—C21	117.9 (3)	N2—C20—N3	112.1 (3)
C19—N1—N2	113.6 (3)	N2—C20—H20	124.0
C19—N1—C21	128.4 (3)	C24—C23—C22	121.1 (3)
C19—N3—C22	126.3 (3)	C28—C23—C24	118.8 (3)
C20—N3—C19	108.9 (3)	C28—C23—C22	120.1 (3)
C20—N3—C22	124.8 (3)	N3—C22—C23	112.5 (3)
C20—N2—N1	102.8 (3)	N3—C22—H22A	109.1
N1—C19—Ir1	128.8 (2)	N3—C22—H22B	109.1
N1—C19—N3	102.6 (3)	C23—C22—H22A	109.1
N3—C19—Ir1	128.6 (2)	C23—C22—H22B	109.1
C12—C7—P1	122.6 (2)	H22A—C22—H22B	107.8
C12—C7—C8	118.0 (3)	N1—C21—H21A	109.5
C8—C7—P1	118.6 (2)	N1—C21—H21B	109.5
Ir1—C32—H32	114.0	N1—C21—H21C	109.5
C33—C32—Ir1	70.37 (19)	H21A—C21—H21B	109.5
C33—C32—H32	114.0	H21A—C21—H21C	109.5
C33—C32—C31	124.1 (3)	H21B—C21—H21C	109.5
C31—C32—Ir1	112.6 (2)	C36—C35—H35A	108.9
C31—C32—H32	114.0	C36—C35—H35B	108.9
C7—C12—H12	119.5	C36—C35—C34	113.2 (3)
C11—C12—C7	121.0 (3)	H35A—C35—H35B	107.8
C11—C12—H12	119.5	C34—C35—H35A	108.9
C2—C1—P1	120.8 (2)	C34—C35—H35B	108.9
C2—C1—C6	118.8 (3)	C12—C11—C10	120.3 (3)
C6—C1—P1	120.4 (2)	C12—C11—H11	119.8
C13—C14—H14	120.0	C10—C11—H11	119.8
C15—C14—H14	120.0	C33—C34—C35	114.4 (3)
C15—C14—C13	119.9 (3)	C33—C34—H34A	108.7
C14—C13—P1	118.2 (2)	C33—C34—H34B	108.7
C18—C13—P1	122.7 (2)	C35—C34—H34A	108.7
C18—C13—C14	119.2 (3)	C35—C34—H34B	108.7
C16—C17—H17	120.2	H34A—C34—H34B	107.6
C16—C17—C18	119.5 (3)	C32—C31—H31A	109.0
C18—C17—H17	120.2	C32—C31—H31B	109.0
C7—C8—H8	119.7	C32—C31—C30	113.0 (3)
C9—C8—C7	120.7 (3)	H31A—C31—H31B	107.8
C9—C8—H8	119.7	C30—C31—H31A	109.0
C2—C3—H3	120.0	C30—C31—H31B	109.0
C4—C3—H3	120.0	Ir1—C29—H29	113.6
C4—C3—C2	120.0 (3)	C36—C29—Ir1	71.98 (18)
C8—C9—H9	119.7	C36—C29—H29	113.6
C10—C9—C8	120.5 (3)	C36—C29—C30	126.3 (3)
C10—C9—H9	119.7	C30—C29—Ir1	109.9 (2)
C1—C2—H2	119.7	C30—C29—H29	113.6
C3—C2—C1	120.6 (3)	C27—C28—C23	119.8 (4)
C3—C2—H2	119.7	C27—C28—H28	120.1
Ir1—C36—H36	114.2	C23—C28—H28	120.1
C35—C36—Ir1	112.2 (2)	C24—C25—H25	120.5
C35—C36—H36	114.2	C24—C25—C26	119.0 (4)
C29—C36—Ir1	71.35 (19)	C26—C25—H25	120.5
C29—C36—H36	114.2	C31—C30—H30A	108.8
C29—C36—C35	123.3 (3)	C31—C30—H30B	108.8
C23—C24—H24	119.2	C29—C30—C31	113.8 (3)
C25—C24—H24	119.2	C29—C30—H30A	108.8
C25—C24—C23	121.5 (3)	C29—C30—H30B	108.8
C14—C15—H15	119.7	H30A—C30—H30B	107.7
C14—C15—C16	120.6 (3)	C27—C26—C25	119.8 (3)
C16—C15—H15	119.7	C27—C26—H26	120.1
C17—C16—C15	120.0 (3)	C25—C26—H26	120.1
C17—C16—H16	120.0	F1—B1—F2	109.1 (3)
C15—C16—H16	120.0	F4—B1—F2	109.9 (3)
C13—C18—C17	120.7 (3)	F4—B1—F1	109.6 (3)
C13—C18—H18	119.6	F4—B1—F3	109.5 (3)
C17—C18—H18	119.6	F3—B1—F2	109.0 (3)
C28—C27—H27	119.5	F3—B1—F1	109.7 (3)
			
Ir1—P1—C7—C12	91.9 (3)	C13—C14—C15—C16	−1.1 (5)
Ir1—P1—C7—C8	−78.1 (3)	C8—C7—C12—C11	2.3 (5)
Ir1—P1—C1—C2	−16.2 (3)	C8—C9—C10—C11	1.8 (5)
Ir1—P1—C1—C6	166.2 (2)	C3—C4—C5—C6	−0.1 (5)
Ir1—P1—C13—C14	−54.1 (3)	C9—C10—C11—C12	−0.7 (5)
Ir1—P1—C13—C18	126.2 (2)	C2—C1—C6—C5	−0.7 (4)
Ir1—C32—C33—C34	101.5 (3)	C2—C3—C4—C5	−0.6 (5)
Ir1—C32—C31—C30	11.3 (4)	C36—C35—C34—C33	−32.2 (5)
Ir1—C36—C35—C34	10.4 (4)	C36—C29—C30—C31	−44.6 (5)
Ir1—C36—C29—C30	101.8 (3)	C24—C23—C22—N3	−48.2 (4)
Ir1—C33—C34—C35	37.2 (4)	C24—C23—C28—C27	0.2 (5)
Ir1—C29—C30—C31	37.2 (4)	C24—C25—C26—C27	0.8 (5)
P1—C7—C12—C11	−167.8 (3)	C15—C14—C13—P1	−177.7 (2)
P1—C7—C8—C9	169.3 (2)	C15—C14—C13—C18	1.9 (5)
P1—C1—C2—C3	−177.6 (2)	C16—C17—C18—C13	0.3 (5)
P1—C1—C6—C5	176.9 (2)	C18—C17—C16—C15	0.5 (5)
P1—C13—C18—C17	178.1 (2)	C33—C32—C31—C30	92.3 (4)
N1—N2—C20—N3	0.1 (4)	C4—C3—C2—C1	0.5 (5)
N2—N1—C19—Ir1	179.9 (2)	C4—C5—C6—C1	0.7 (5)
N2—N1—C19—N3	1.0 (3)	C6—C1—C2—C3	0.1 (5)
C19—N1—N2—C20	−0.7 (3)	C20—N3—C19—Ir1	−179.8 (2)
C19—N3—C20—N2	0.5 (4)	C20—N3—C19—N1	−0.9 (3)
C19—N3—C22—C23	−81.1 (4)	C20—N3—C22—C23	99.2 (4)
C7—P1—C1—C2	−139.5 (3)	C23—C24—C25—C26	1.0 (5)
C7—P1—C1—C6	42.9 (3)	C22—N3—C19—Ir1	0.5 (4)
C7—P1—C13—C14	72.6 (3)	C22—N3—C19—N1	179.4 (3)
C7—P1—C13—C18	−107.0 (3)	C22—N3—C20—N2	−179.8 (3)
C7—C12—C11—C10	−1.4 (5)	C22—C23—C28—C27	−178.9 (3)
C7—C8—C9—C10	−0.8 (5)	C21—N1—N2—C20	−176.9 (3)
C32—C33—C34—C35	−45.2 (5)	C21—N1—C19—Ir1	−4.4 (5)
C32—C31—C30—C29	−32.4 (4)	C21—N1—C19—N3	176.7 (3)
C12—C7—C8—C9	−1.2 (5)	C35—C36—C29—Ir1	−104.8 (3)
C1—P1—C7—C12	−145.1 (3)	C35—C36—C29—C30	−3.0 (5)
C1—P1—C7—C8	44.9 (3)	C31—C32—C33—Ir1	−104.6 (3)
C1—P1—C13—C14	−179.9 (2)	C31—C32—C33—C34	−3.1 (5)
C1—P1—C13—C18	0.5 (3)	C29—C36—C35—C34	92.1 (4)
C14—C13—C18—C17	−1.5 (5)	C28—C27—C26—C25	−2.1 (5)
C14—C15—C16—C17	−0.1 (5)	C28—C23—C22—N3	131.0 (3)
C13—P1—C7—C12	−36.7 (3)	C25—C24—C23—C22	177.7 (3)
C13—P1—C7—C8	153.3 (2)	C25—C24—C23—C28	−1.5 (5)
C13—P1—C1—C2	111.7 (3)	C26—C27—C28—C23	1.5 (5)
C13—P1—C1—C6	−65.9 (3)		

### Hydrogen-bond geometry (Å, º)

**Table:**

*D*—H···*A*	*D*—H	H···*A*	*D*···*A*	*D*—H···*A*
C20—H20···F2^i^	0.95	2.29	3.131 (4)	147

Symmetry code: (i) −*x*+1, −*y*+1, −*z*.
